# Public finances and tobacco taxation with product variety: Theory and application to Senegal and Nigeria

**DOI:** 10.1371/journal.pone.0212015

**Published:** 2019-02-14

**Authors:** Théophile T. Azomahou, Racky Baldé, Abdoulaye Diagne, Pape Yona Mané, Ibrahima Sory Kaba

**Affiliations:** 1 University Clermont Auvergne, CNRS, IRD, CERDI, Clermont, France; 2 Maastricht University, School of Business and Economics, Maastricht, The Netherlands; 3 United Nations University (UNU-MERIT), Maastricht, The Netherlands; 4 Consortium pour la Recherche Economique et Sociale (CRES), Dakar, Senegal; University of Illinois at Chicago, UNITED STATES

## Abstract

This study endeavors to answer two questions: which category of excise taxes is more appropriate for Senegal and Nigeria and which consequences an increase of the tobacco taxes would have on the price, the demand and the tax revenues in each one of the two countries? To answer these questions, we adopt a double approach: first, a theoretical model of taxation with variety; and second, a simulation model to answer the second question. The results of the theoretical model indicate that, in the context of excise taxation, the number of products variety—or that of cigarette brands—directly affects both the degree of market concentration and the marginal effects of specific and ad valorem excise taxes on the price of tobacco. In addition, the comparison of the marginal effects of ad valorem and specific excise taxes depends on the marginal costs of production of different varieties weighted by the tax rates and the number of varieties. Our empirical results first show that the specific excise taxes are more adapted to Senegal while ad valorem excise taxes fit best Nigeria. This result crucially matters for the excise taxes are exclusively of an ad valorem nature in both Senegal and Nigeria. It is perfectly possible to envisage a situation where the two main forms of excise taxes could co-exist. It also appears from our results that tax development does not have the same implications for the two countries. Increasing tobacco taxes in Senegal strongly reduces the demand, but also induces a decrease in the tax revenues, while this will imply a lesser decline in demand in Nigeria accompanied however by a sharp increase of the country’s tax revenues. This difference stems from the fact that the price-elasticity of tobacco demand is very high in Senegal, contrary to Nigeria. Finally, it is important to mention that there is a specific threshold beyond which the tax increases cease to have a positive effect on tax revenues in Nigeria.

## Introduction

The use of excise taxes—ad valorem and specific—as a means to control cigarettes consumption is a relatively new toolkit in many developing countries ([1]). There is a long and documented history throughout the world of governments implementing such tax schemes to generate revenues. In many instances, tobacco taxation has also been highlighted as an efficient means of mobilizing domestic resources to finance health and other important development programmes ([2]). The recent settings of the Addis Ababa Action Agenda and the 2030 United Nations’ Agenda for Sustainable Development have further heightened the ever growing interest in tobacco taxation. More specifically, the latest Agenda intends to strengthen the country-level implementation of the World Health Organization’s (WHO’s) Framework Convention on Tobacco Control (FCTC). This framework convention is an international treaty, ratified by nearly 180 countries that pledged to protect public health by setting up concrete measures to control the tobacco use. Article 6 of the FCTC recognizes price and tax measures as effective means to reduce the demand for tobacco, and the guidelines for Article 6’s implementation encourage the use of taxation in comprehensive strategies for tobacco control.

Tobacco taxation could also be used as an effective and important evidence-based vehicle to help many countries achieve their long-term development objectives ([3]). As tobacco tax rates in many low- and middle-income countries are currently low and demand for tobacco products is relatively inelastic, many countries could increase government revenues substantially through tobacco taxation ([4]). By creating the fiscal space to finance development programmes while, at the same time, reducing tobacco use, tobacco taxation could indeed be a win-win policy for governments throughout the world.

An African country provides a recent example of a government-led aggressive anti-tobacco strategy. In November 2013, President Ian Khama of Botswana in his yearly State of the Union address, announced a 30% increase in tobacco custom taxes in addition to the already agreed-upon 48% excise taxes scheme by the five member states of the Southern Africa Customs Union (SACU). Another example comes from the Philippines, where the tax on low-priced brands of cigarettes was increased by 341%, which in turn led to a 114% increase in annual excise revenue ([2]). Under these reforms, 85% of the extra tax revenue is being used to subsidize universal health care of 14 million families and upgrade medical facilities. This example of the Philippines shows that a substantial increase of tobacco taxation could lead to an equally improvement of public health and tax revnues. There are at least 30 other countries that dedicate a certain amount of their tobacco taxes on health. Although such dedicated allocations are not always feasible, the reforms in the Philippines have convincingly shown that substantial increases in tobacco taxation can lead to improvements in public health finance. Many retrospective studies have shown the very importance of tobacco taxation in public health outcomes. For instance, it has been observed by [5] amongst others that a 10% increase in cigarette taxes in the United States could potentially decrease the number of deaths from respiratory cancers by up to 1.5%. The French government increased cigarettes taxes substantially from the mid-1990s, with cigarette prices tripling in real terms by 2005. Amongst French males, rates of death from direct lung cancers fell by roughly 50% during the same period ([6, 7]).

A remarkable anomaly of the current literature of tobacco comes from the very limited amount of empirical research on developing countries—especially on Africa—while the bulk of the previous empirical studies have heavily focused on the industrialized world ([8]). The very first empirical study on the impact of taxation on tobacco demand dates back to [9], who used data from 1973 to 1986 to estimate excise-tax elasticity of demand in Papua New Guinea. Amongst the many reasons of the scarcity of research on developing countries, the lack of sufficiently reliable data is often mentioned. This research contributes to that effort.

Relying primarily on the World Health Organization’s Global Adult Tobacco Survey (GATS) and on the results derived from our previous work in [10], this research intends to: (i) determine which category of excise taxes is more appropriate to the market of cigarettes in Senegal and in Nigeria; (ii) analyze the impact on prices, demand and tax revenues, of an increase of tobacco taxes for the two aforementioned countries. To do so, we first propose a theoretical model of taxation of the cigarette market in Senegal and in Nigeria in order to understand which category of excise taxes—ad valorem or specific—fits best the local context. This model is an extension of [11] and [12], who themselves are based on [13]. Our model markedly differs from those by the introduction of product variety, with a direct application to the tobacco market. The results of the theoretical model show that in the context of excise taxation, the number of brands directly affects the degree of market concentration and the marginal effects of ad valorem and specific excise taxes on the price of tobacco. In addition, the ratio of the marginal effects of the ad valorem and specific excise taxes depends on the marginal costs of production of the different varieties weighted by the tax rates and the number of product varieties. The model shows that specific excise taxes are more appropriate to Senegal while ad valorem excise taxes fit best Nigeria. This result matters for policy given the fact that excise taxes are exclusively ad valorem by design in Senegal and in Nigeria.

Furthermore, we build on a simulation model of cigarettes taxation, previously used at various degrees by [4] and [2], among others. The [2] model is a global model of the tobacco market, which builds on data originating from 181 countries, hence accounting for nearly 98% of the world’s smokers in 2014. More specifically, we estimate the relative variations of prices, demand and tax revenues as well as the critical taxation thresholds stemming from a continuous increase of excise taxes. The very low levels of taxation in Senegal and Nigeria indicate important margins for their respective governments. For instance, the rate of excise taxes for the most consumed brand of cigarettes in Senegal and Nigeria in 2016 was 22.5 and 15.87% respectively ([14]). The overall levels of taxation for the most popular brand of cigarettes in Senegal and in Nigeria was 37.75 and 20.63% respectively. Throughout the simulation, we borrow extensively from [10] on their estimations of the price-elasticities of demand in Senegal and in Nigeria. It also appears that tax development does not have the same overriding implications for the two countries. Increasing taxes on tobacco in Senegal and in Nigeria drastically reduces its demand, but also implies a sharp decline in the revenues for the former and and a rise of tax revenues for the latter. This stark difference stems from the fact that the price-elasticity of tobacco demand is very high in Senegal (-1.29) while it is very low in Nigeria (-0.217). Finally, it is important to notice that there exists a certain threshold beyond which tax increases cease to have a positive effect on tax revenues in Nigeria. This simulation model constitutes an additional contribution to the literature of addictive goods in developing countries in general and in Africa in particular.

The remainder of the analysis is organized as the following. Section 2 discusses the literature on tobacco taxation. Section 3 describes the underlying theoretical model that supports the analysis of the market concentration of tobacco in Senegal and Nigeria. Section 4 presents the simulation model allowing to evaluate the impact of hypothetical increases of taxes on the price, the demand and the tax revenues. Section 5 describes the results, and section 6 concludes.

## Taxation of tobacco: A literature review

The main historical reason for tobacco and tobacco products taxation remains its enormous potential to generate substantial revenues for states. While tax revenues have at point in time represented up to 5% of developed countries’ total tax revenues, their importance has never since ceased to decline in those countries. Contrarily, the revenues generated by the taxation of tobacco products represent currently important shares of the revenues perceived by emerging and developing countries ([15]). Tobacco taxes are also the best tools to control and promote cigarettes cessation, prevention and the reduction their consumption ([5, 15]). Several empirical studies in developed countries have shown in a rather quite convincing manner that the increase of prices induced by a significant increase of taxes leads to an important reduction in cigarettes consumption ([2, 12]). Equivalently, a growing number of empirical studies on developing countries suggests that an increase of prices leads to a more drastic reduction of cigarettes consumption ([16]).

### Taxes, price and tobacco demand

For a long period of time, the idea that cigarettes and other addictive goods were the exceptions to the classic law of demand, was the dominant paradigm ([17–19]), suggesting—ceteris paribus—that the consumption of a given good decreases once its price increases. Recently, many studies have proven that the demand of the tobacco products is in fact sensitive to price variations, as well as to those of other factors ([15]). The bulk of the empirical estimations of the price-elasticity of tobacco demand in developed countries vary between -0.25 and -0.50, with a strong concentration around -0.40. In addition, the estimates of the price-elasticity of demand in developing countries range between -0.50 and -1.00 for a large section of the specialized literature ([20]).

The most recent studies in developed countries have used micro-level data on individual cigarettes consumption, and have lead to conclude that nearly more than half of the effect of the price on demand affects smoking prevalence while the other half affects the demand of the early smokers ([21–24]). Some studies have found that young smokers are more sensitive than adults to changes in the price of tobacco, and have therefore concluded towards an inverse relationship between the price-elasticity of tobacco demand and the age of the users, with teenagers more than three times sensitive to price hikes ([22, 25]). Other studies are not entirely in accordance with those conclusions. For example, [24] have found that young people are no more sensitive to cigarettes price variations than the adults.

Regarding the developing world, the number of studies is not that remarkable. The mainstream economic theory for very long considered that the demand of cigarettes in developing countries is more sensitive to changes in price ([26]). The first to estimate the impact of taxes on tobacco demand in a developing country were [9]. Using data on tobacco and its various products in Papua New Guinea, from 1973 to 1986, they found estimates of price-elasticities of -0.71 for tobacco and of -0.50 for other tobacco products. Like [26], their estimates under-estimate the true value of the price-elasticity of demand given that all tax increases are not always entirely passed on to prices. In supposing that taxes represent more than half of the prices, then the price-elasticity of tobacco demand and that of other tobacco products get equal to -1.42 and -1.00 respectively. The estimations of [9] provide the first hand-on evidence that the demand of tobacco products in developing countries is more elastic to price variations than in developed countries.

Some authors such as [27] reached rather a different set of conclusions. Relying on time series of cigarettes demand in Turkey, from 1960 to 1988, the author found that the short-term price-elasticity of demand was equal to -0.21 while the long-term price-elasticity of demand was in the vicinity of -0.37. Many other studies have examined the price-elasticity of cigarettes demand in China ([28–30]). Those elasticities, mostly centered around a -0.75 value, are coherent with the hypothesis that the demand of cigarettes in developing countries is more sensitive to price variations than in developed countries. However, it is worth pointing out the potential lack of variations in prices in a country like China, a feature that might be at work in African countries as well. [16] and [4] have examined the impact of taxation on tobacco demand and tax revenues in developing countries. [4] provide the very first tax simulation of tobacco products in Cameroon.

There are very few studies that take into account all countries altogether, notwithstanding the income levels and the geographical location. [2]’s is an exception in that regard, focusing on all continents with various income levels.

### Ad valorem or specific excise taxes?

The debate on the advantages and disadvantages of specific excise taxes compared to ad valorem excise taxes—either applied or not to tobacco products—is not new at all. In fact, this debate is as old as the discipline of public finance itself. The equivalence of these two tax regimes under pure and perfect competition—a rather rare case—is a stylized fact in the literature. The realization that these two tax regimes necessitate different treatments in the absence of pure and perfect competition took roots with [31]. The analysis of the monopolistic situation continued with [32]—who has shown that the specific excise taxation allows to generate further revenues than the ad valorem excise taxation—and culminated with [33]. The debate has never stopped ever since and continues to attract the attention of both academics and policymakers. [11], while comparing the specific excise taxation to the ad valorem excise taxation in an oligopolistic context, with and without free entry, have shown that the first dominates the second when the overall general welfare is concerned.

### Models of taxation

The [13] model is one of the pioneers in the analysis of the effects of taxation, of price control and of public procurements on prices, of the sectorial production and of the firms’ profits in a market with different possible structures: pure and perfect competition, monopoly and oligopoly à la Cournot. In this model, the possible government interventions could take various forms: different categories of taxes, combination of taxes and regulation policies. The first contribution of [13] was the introduction of monopolistic competition as a perfect equilibrium in a market where the entry decisions of firms are essentially motivated by their ex-post profits. This allowed [13] to pursue the analysis of the effects of excise taxes [34] in an oligopolistic framework with a certain number of firms to a situation of monopolistic competition. The comparison in [13] of different market structures shows that the positive effect of taxes on the price would be further important in a monopolistic competition framework only in the situation where taxes reduce the profits of a certain number of firms. In addition, when the price-elasticity of demand is very low, the excise taxes could eventually increase the profits of the firms.

The theoretical framework of [13] is the starting point of the [11]’s model, which focuses on a systematic comparison of the effects of marginal ad valorem and specific excise taxes on the price in the intermediary and more realistic context of imperfect competition, away from the extreme scenarios of pure and perfect competition and of monopolistic competition with or without free entry. Their comparative statics show a superiority of ad valorem excise taxes on the specific excise taxes when the welfare of the consumer is concerned (under the condition that free entry is not possible). The immediate implications are a relatively low price, higher tax revenues and lower profits for the industry in general. [12] are in a way the empirical vindication of the [11]’s model. They investigate the question of tobacco tax harmonization in Europe in a taxation model heavily borrowing from [11]. They mostly provide an estimation from a price-cost mark-up reduced-form model of the tobacco market in 12 European countries between 1982 and 1997.

## Theoretical model

The theoretical model presented here is a further modification of [11] and [12], who themselves are two extensions of [13]. The commonality between all these approaches is the underlying hypothesis about the very nature of the good traded: it is considered as unique and totally homogeneous. Our main contribution consists of considering a unique good with different varieties—which perfectly corresponds to the case of cigarettes—drawing from [35]. This is equivalent of assuming that each firm produces its own brand. But before presenting the baseline model we will briefly present a couple of descriptive statistics about the tobacco markets in Senegal and Nigeria.

### Analysis of market concentration

The GATS provides six official brands of cigarettes in Senegal and eight brands in Nigeria. The six brands in Senegal are: Excellence, Houston, Marlboro, Dunhill, Davidoff and Gold Seal. Of all those brands only the first three are consistently and mostly reported by the consumers interviewed in the survey. Other brands, such as Camelia, L&M, and Gauloises are also present on the market, though were not taken into account in the analysis, mainly because their consumption was quite marginal. All these brands, either mentioned in the analysis or not, have in common to be international. The brands in Nigeria are: Aspen, Benson and Hedges, Consulate, Dunhill, Gold Leaf, London White, Rothmans and Standard. Each brand has a specific market share, which depends on the average price of the brand, on its advertizing efforts, as well as on other relevant parameters. Denoting by *s*_*i*_ the market share of the *i*^*th*^ brand, the ratio of market concentration is defined as:

$$\begin{array}{c} CR_{m}=\sum_{i=1}^{m}s_{i} \end{array}$$ (1)

As it is common practice in the relevant literature, we will fix *m* in the previous equation to be equal to four. The corresponding value of the market concentration ratio is referred to as “the Four-Firm concentration ratio”. *CR*_4_ denotes therefore the total market share of the biggest four brands of tobacco in Senegal and Nigeria. This measure of market concentration varies between 0 and 100, and depending on its contemporaneous value, we talk of *no*, *weak*, *average*, *strong* or of *total* market concentration.

**Fig 1:**
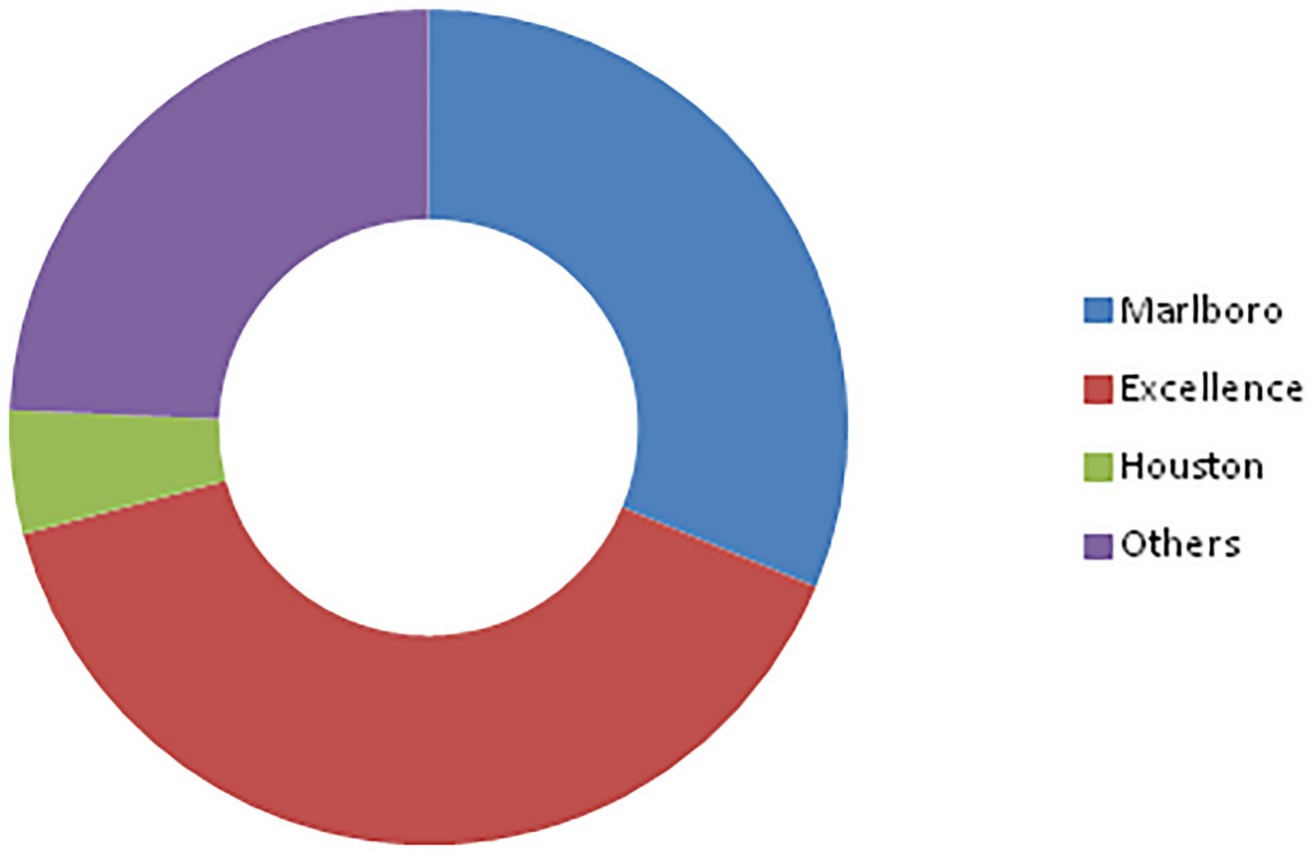
Market shares of cigarettes in Senegal. Each color represents a specific brand of cigarette. The data used comes from the GATS.

**Fig 2:**
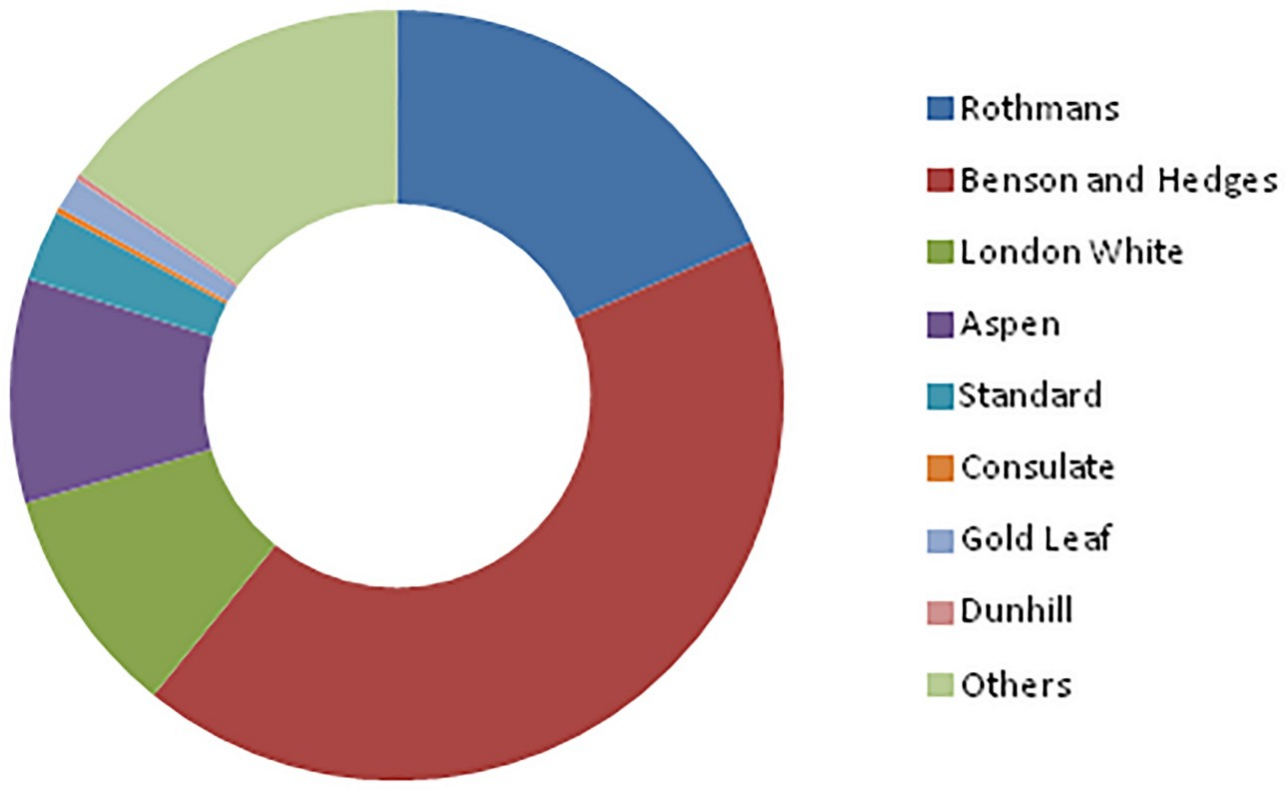
Market shares of cigarettes in Nigeria. Each color represents a specific brand of cigarette. The data used comes from the GATS.

Because the GATS provides the market shares for only the three most important tobacco brands for Senegal, we could only compute the concentration index for the two biggest brands, or *CR*_2_. A simple analysis of the market concentration ratio *CR*_2_ in Senegal shows a rather strong market concentration of cigarette in the country. In fact, the two most purchased brands in 2014—Excellence and Marlboro—represent together nearly 70% of the market shares. This resembles a duopoly, either by design or by accident. Looking at the data inherent to Nigeria almost brings similar observations: the market of cigarette in Nigeria is an oligopoly, where the four biggest brands share almost 80% of the market. Figs 1 and 2 below show the market shares of different brands of cigarette in Senegal and in Nigeria, respectively. They provide a visual overview of the market concentration of tobacco in the two countries.

Because it does not internalize the market shares of all the firms within a given industry, the concentration ratio therefore does not provide a clear idea of the firms’ size distribution. It also does not provide further details on the competitiveness of the industry. *CR*_4_ only gives an idea on the oligopolistic nature of the tobacco industry while offering at the same time a concrete measure of the market concentration. To curtail some of these imperfections and shortcomings, we will evaluate the Herfindahl Index of the tobacco industry in Senegal and in Nigeria. This index (also referred to as the Herfindahl-Hirschman Index, or simply HHI) is a measure of the relative size of the firms in relation with the relevant industry and is a reliable indicator of the degree of competition among them. For a market share *s*_*i*_ of the *i*^*th*^ firm or of the *i*^*th*^ brand, the Herfindahl-Hirschman Index is defined as the following:

$$\begin{array}{c} H=\sum_{i=1}^{N}s_{i}^{2} \end{array}$$ (2)

where *N* is the total number of firms or rather the total number of brands in our specific case. *H* varies between 1/*N* and 1, and an increase of *H* implies a high degree of market concentration while a lower value shows a highly competitive market.

Using the market shares provided by the GATS, the computed Herfindahl index amounts to 31.64% for Senegal and to 25.62% for Nigeria. These numbers confirm the conclusions induced originally by the market concentration ratio indices, with perhaps a greater clarity: (a) the market of cigarette is highly concentrated both in Senegal and in Nigeria; (b) this concentration is more emphasized in Senegal than in Nigeria. These observations however only provide a glimpse of what might be more dynamic tobacco markets, providing that they rely exclusively on the GATS. As such, they should not be taken as the absolute description of the cigarettes markets in Senegal and Nigeria.

### Baseline model

The industry produces the same good in different varieties. The number of varieties corresponds to *m*(*m* ≥ 2). The number of varieties—or of brands in the case of cigarettes—could be identical to the number of firms present on the market. The quantity produced and sold of variety *i* of the good is denoted by *x*_*i*_. The production cost *C*(⋅) of variety *i* of the good depends on the quantity produced of that good, i.e., *C*(⋅) ≡ *C*(*x*_*i*_). This function is positive and possesses increasing returns to scale, i.e., $C_{x_{i}}\left(x_{i}\right)x_{i}/C\left(x_{i}\right)<1$. The marginal cost of production is $C_{x_{i}}\left(x_{i}\right)\equiv dC\left(x_{i}\right)/dx_{i}$. The total quantity produced of the good by the industry is given by the following function:

$$\begin{array}{c} X=(\int_{0}^{m}x_{i}^{\alpha }di{)}^{1/\alpha } \end{array}$$ (3)

where *α* ∈ (0, 1] represents the degree of substitution between different varieties of the good. This formulation is a generalization of the product variety model of [35]. Another formulation of this function is proposed by the Cobb-Douglas production function of [36], with:

$$\begin{array}{c} Y_{t}={\left(A_{t}L_{t}\right)}^{1-\beta }(\sum_{0}^{m}x_{i}^{\alpha }di{)}^{\beta /\alpha } \end{array}$$ (4)

where *A*_*t*_ and *L*_*t*_ are the total factor productivity (TFP) and labor, while *β* is the contribution of all other factors different from labor to the final production *Y*_*t*_. To make the computations tractable and without any loss of generality, we will consider in the remainder of the analysis that *α* ≈ 1, suggesting that the different varieties of the good are highly substitutable. The composite price *P*(*X*) of the good is a function of the aggregate production of the industry, and is an inverted function of the demand, i.e., the partial derivative of *P*(*X*) denoted by *P*_*X*_(*X*) is such as *P*_*X*_(*X*) < 0. The price-elasticity of demand of the industry is measured by *e*_*PX*_ = −*P*(*X*)/*XP*_*X*_(*X*) > 0. The excise tax is made of ad valorem and specific taxes, with the respective rates of *t*_*s*_ and *t*_*v*_. Taking into account all these ingredients, the profit associated to the production of the variety *i* of the good is therefore:

$$\begin{array}{c} \pi_{i}=[\left(1-t_{v}\right)P\left(X\right)-t_{s}]x_{i}-C\left(x_{i}\right) \end{array}$$ (5)

The quantity *dX*/*dx*_*i*_ = λ represents the strategic interaction between the producers of different varieties of the good. This quantity could take many values. For λ = 1, the optimization problem corresponds to a Cournot conjecture. For λ = 0, the market is completely “competitive” and the optimization problem therefore boils down to a Bertrand conjecture, where the equilibrium composite price of the good corresponds to the marginal cost. Finally, for λ = *m*—meaning that the total number of varieties −, the conjecture of the market corresponds to situation of “tacit collusion”, which indeed corresponds to the situation we envisage in the remainder of this analysis.

The first-order derivative of the profit function corresponds to:

$$\begin{array}{c} \frac{d\pi_{i}}{dx_{i}}=\left(1-t_{v}\right)P\left(X\right)-t_{s}+\left(1-t_{v}\right)mP_{X}\left(X\right)x_{i}-C_{x_{i}}\left(x_{i}\right)=0 \end{array}$$ (6)

where *P*_*X*_ and $C_{x_{i}}$ are the partial derivatives. By dividing this equation by *m* and by taking the integral on the interval [0, *m*], we get:

$$\begin{array}{c} \left(1-t_{v}\right)P\left(X\right)[1+\frac{P_{X}\left(X\right)}{P(X)}X]-\frac{1}{m}\int_{0}^{m}C_{x_{i}}\left(x_{i}\right)di-t_{s}=0 \end{array}$$ (7)

Solving this problem with respect to the composite price *P*(*X*) and by using the fact that the price-elasticity of demand of the industry *e*_*PX*_ ≡ *e* = *P*(*X*)/*XP*_*X*_(*X*) > 0, we get:

$$\begin{array}{c} P\left(X\right)=\frac{1}{1-\frac{1}{e}}\underset{\varphi (m)}{\underset{︸}{\frac{1}{1-t_{v}}(\frac{1}{m}\int_{0}^{m}C_{x_{i}}\left(x_{i}\right)di+t_{s})}}=\frac{1}{1-\frac{1}{e}}\varphi \left(m\right) \end{array}$$ (8)

*φ*(*m*) could be considered as the sum of all the marginal costs—for each variety of the good—weighted by the ad valorem and specific excise taxes. Relying on the tools of comparative statics, we get the following results:

**Proposition 1** *In an excise taxation model with different varieties (brands)*:

1. *The specific and ad valorem taxes affect the composite price of the good such as*:
  $$\begin{array}{c} \frac{dP(X)}{dt_{s}}=\frac{1}{\left(1-t_{v}\right)\left(2+A-E\right)}>0 \end{array}$$ (9)
  *and*
  $$\begin{array}{c} \frac{dP(X)}{dt_{v}}=\varphi \left(m\right)\frac{dP(X)}{dt_{s}}>0 \end{array}$$ (10)
  *where* $A=-\frac{\int_{0}^{m}C_{x_{i}x_{i}}\left(x_{i}\right)di}{m^{2}\left(1-t_{v}\right)P_{X}\left(X\right)}$ *and* $E=-\frac{P_{XX}\left(X\right)X}{P_{X}\left(X\right)}$.
2. *The number of varieties m directly affects both the marginal effects of the specific and the ad valorem excise taxes on the composite price of the market*.

**Proof 1** Eq (9) *derives from the application of the implicit function theorem to* Eq (7); *while* Eq (10) *derives from the observation that P*(*X*) + *XP*_*X*_(*X*) = *φ*. *In addition*, *E denotes the elasticity of the slope of the inverse demand, as defined by* [12]. *The second part of the proposition derives from the fact that* $\varphi \equiv \varphi \left(m\right)=\frac{1}{1-t_{v}}(\frac{1}{m}\int_{0}^{m}C_{x_{i}}\left(x_{i}\right)di+t_{s})$. *Inequality* (9) *results from supposing that the stability condition of* [37] *holds, a restriction somewhat stronger than the second order condition and also used by* [11] *and* [12]. *This refers to the case in which the number of firms is fixed as in the Generalized Cournot model and to that in which entry and exit are free, as in the Free Entry Oligopoly. As a result, we get that*: 2 + *A* − *E* > 0.

By comparing the effects of the specific and ad valorem excise taxes on the composite price of the good and by using the expression of this price in Eq (8), we get the following proposition:

**Proposition 2** *In an excise taxation model with different varieties (brands)*:

1. *The comparative statics of the effects of specific and ad valorem excise taxes differ in function of the weighted value of the marginal costs φ, and therefore of the number of varieties m*:
  $$\begin{array}{c} \frac{\frac{dP(X)}{dt_{s}}}{\frac{dP(X)}{dt_{v}}}=\frac{1}{\varphi (m)} \end{array}$$ (11)
2. *The degree of market concentration (μ) derives from the effects of specific and ad valorem excise taxes, and is a function of solely the price-elasticity of demand*:
  $$\begin{array}{c} \mu \left(e\right)=\frac{\frac{dP(X)}{dt_{s}}}{(\frac{1}{P(X)})\frac{dP(X)}{dt_{v}}}=\frac{1}{1-\frac{1}{e_{PX}}}=\frac{P(X)}{\varphi (m)} \end{array}$$ (12)

**Proof 2** Eq (11) *derives from the simple manipulation of the effect of the ad valorem excise taxes on the composite price from* Eq (10). *The degree of market concentration is obtained by dividing the composite price in* Eq (8) *by the weighted marginal cost φ*(*m*).

The degree of market concentration *μ*—which also encapsulates the mark-up—is the key parameter in the remainder of our analysis. In the situation of pure and perfect competition—an extreme case—*μ* = 1 and corresponds to the situation where the marginal effects of the specific and ad valorem excise taxes are equivalent. In the more realistic case where *μ* > 1, this implies that the effect of the specific excise taxes on the price exceeds that of the ad valorem excise taxes, to a value corresponding to the mark-up. In what follows, we rely on the empirical results of [10], who offer a reasonable approximation of various price-elasticities of tobacco demand in both Senegal and Nigeria, to try answer the first question of this analysis.

The sample from the GATS contains individuals aged 15 or more, with 4343 individuals for Senegal and 9705 for Nigeria. The survey also provides various information on the socio-economic characteristics of the individuals, tobacco consumption, smokeless tobacco use, smoking cessation, second-hand smoke, manufactured cigarettes, media knowledge, attitudes as well as perceptions. The estimation strategy followed in [10] follows the Heckman selection model, from the original [38]. This estimation strategy evaluates two equations: a selection equation—a Latent Probit model—and an intensity equation of cigarettes consumption. [10] provide further details about the methodology, the variables’ description as well as the overall results. Using the Full Information Maximum Likelihood (FIML) approach to estimate the intensity equation, the authors get -1.29 and -0.217 as the aggregate price-elasticities of cigarettes demand for Senegal and Nigeria respectively. Plugging these price-elasticities in the absolute value of the mark-up gives 4.44 and 0.27 respectively. A mark-up of 4.44 shows that the marginal effect of the specific excise taxes on the price dominate that of the ad valorem excise taxes in Senegal, while a mark-up of 0.27 indicates that the marginal effect of the specific excise taxes is dominated by that of the ad valorem excise taxes in Nigeria. Therefore the specific excise taxes seem more appropriate for Senegal while the ad valorem excise taxes seem to be a better fit for Nigeria.

## Simulation model

To estimate the effect of an increase in excise taxes on the price, the demand of cigarettes and level of tax revenues is the logical follow-up of the theoretical model. The simulation model relied upon here heavily borrows from [4] and [2]. The key ingredient of the model is the price-elasticity of demand. The underlying hypothesis of the aforementioned models is that all successive increases of the tobacco taxes are entirely reported on the price. Although common, that assumption seems too heroic, especially in a developing country context. We will assume instead that ‘only’ 75% of the excise tax increases—both types—are passed onto the price.

### Taxation and price

The excise tax could be a fixed quantity per pack of cigarettes or a percentage of the value of the pack or simply the combination of the two. For both Senegal and Nigeria, as well as dozens of other countries, the *World Health Organization’s Report on the Global Tobacco Epidemic 2017* provides the taxation figures of the most popular brand of cigarette in 2016. More specifically, this dataset informs on the average price per pack of 20 cigarettes for a wide range of countries on each continent, the levels of excise taxes, the value added taxes as well as other levels of taxation. For instance, the report shows that the overall taxes, taken as a percentage of price of the most sold brand in Senegal and Nigeria, were 37.75% and 20.63% respectively. We will use these these information as our initial baselines to evaluate the effects of an increase of taxes on the demand of cigarettes and on the tax revenues by simulating the successive hypothetical increases of these taxes. Alongside the excise tax, there are two other types of taxes in the dataset: the value added tax (VAT) and the other forms of consumption taxes.

### Taxation and consumption

The amplitude with which an increase of the price of cigarette reduces its consumption heavily depends on the magnitude of the price-elasticity of demand. For example, an elasticity of -0.4 implies that a 10% increase of tobacco price would lead to a 4% reduction of its consumption. The price-elasticity of demand reflects a combination of the conditional demand—i.e., the quantity or the intensity of cigarettes consumption—and the tobacco prevalence. These price-elasticities of demand vary according to households’ income. The most recently robust estimates range the price-elasticity of tobacco demand in developed countries between -0.25 and 0.5, while this elasticity is more than often comprised between -0.2 and -0.8 in developing countries ([2, 39]). These numbers tend to indicate that consumers in developing countries are generally more sensible to price variations than their counterparts in developed countries, for reasons that are essentially inherent to their income levels.

Denoting by *S* the number of packs of cigarettes sold in the retail market, the number of packs sold (S*) in response to a price increase could be calculated as the following:

$$\begin{array}{c} S^{*}=S\times \left(1+\Delta P\times \epsilon_{P}\right) \end{array}$$ (13)

where Δ*P* is the percentage change in terms of retail price and where *ϵ*_*P*_ represents the price-elasticity of demand. This elasticity is the best available variable to evaluate the effects of price increases on consumption. We will rely on the price-elasticity already obtained by [10]. The price-elasticity of demand *ϵ*_*p*_ measures the percentage variation of the quantity demanded of a good when its price varies by 1%. The estimation of this parameter requires a specification of the tobacco demand’s function, and to estimate it using an appropriate methodology. With a price-elasticity of cigarettes demand *ϵ*_*p*_, it is possible to simulate the scenarios from which policymakers from Senegal and Nigeria could rely upon in order to design different fiscal schemes to reduce cigarettes consumption, while keeping to the extent possible the tax revenues from tobacco taxation at decent levels.

The baseline hypothesis made here—as previously stated—is that any one unit increase of the taxes induces a 0.75 unit increase of the prices, and is mostly supported by the consumers. By assuming that the value added taxes and that the other consumption taxes remain unchanged from one period to the other, this suggests that a one unit increase *τ*% of the excise tax is equivalent to a 0.75Δ*P* of the retail price. This also means that the final demand induced by this tax increase corresponds to a quantity *ϵ*_*p*_ × Δ*P*, or simply to the cross-product of the price-elasticity of demand and the price variation.

### Taxation and tax revenues

Taking into account all the previous information on the price and the demand, the relative variation of tax revenues generated is given by:

$$\begin{array}{c} {}[1+(1+\Delta P)\epsilon_{p}]\Delta P \end{array}$$ (14)

This quantity derives from the difference between the tax revenues from one year to the other. Tables 1,2,3 and 4 give the results of different simulations of the price, the demand and the tax revenues, as a consequence of successive increases of tobacco taxes for both Senegal and Nigeria. A simple optimization of Eq (14) suffices to show that the optimal tax increase needed to help maximize the revenues is the following:

$$\begin{array}{c} \Delta^{*}P=-\frac{1+\epsilon_{p}}{2\epsilon_{p}} \end{array}$$ (15)

In the same token, it is possible to evaluate the increase of prices—and that of the taxes—that could help drastically reduce the demand of cigarettes, while keeping unchanged the level of tax revenues with respect to their contemporaneous values. We will denote this critical value by Δ^*o*^ *P*. Beyond this critical threshold, any additional increase of the prices will automatically induce a decline in tax revenues. More specifically, it corresponds to the value of Δ*P* for which the increase of the tax revenues is brought to zero. It is easy to observe that Δ^*o*^ *P* = 2 × Δ* *P*.

**Table 1:** Simulated effects of hypothetical excise tax increases in Senegal and Nigeria for all smokers.

Increase of Prices (Increase of Taxes)	Relative Decline of the Demand		Relative Change of Tax Revenues	
	Senegal	Nigeria	Senegal	Nigeria
5 (6.66)	-6.45	-1.08	-1.77	3.86
10 (13.33)	-12.90	-2.17	-4.19	7.61
15 (20)	-19.35	-3.25	-7.25	11.25
20 (26.66)	-25.80	-4.34	-10.96	14.79
25 (33.33)	-32.25	-5.42	-15.31	18.21
30 (40)	-38.70	-6.51	-20.31	21.53
35 (46.66)	-45.15	-7.59	-25.95	24.74
40 (53.33)	-51.60	-8.68	-32.24	27.84
45 (60)	-58.05	-9.76	-39.17	30.84
50 (66.66)	-64.50	-10.85	-46.75	33.72
60 (80)	-77.40	-13.02	-63.84	39.16
70 (93.33)	-90.30	-15.19	-83.51	44.17
77.51 (103.34)	-100	-16.81	-99.97	47.65
80 (106.66)	…	-17.36	…	48.75
90 (120)	…	-19.53	…	52.89
100 (133.33)	…	-21.70	…	56.60
…	…	…	…	…
180.41 (240.54)	…	-39.14	…	70.63
…	…	…	…	…
360.82 (481.09)	…	-78.29	…	0.00

All the figures in the table are expressed in percentage terms. Price-elasticities of demand: -1.29 for Senegal and -0.217 for Nigeria.

**Table 2:** Simulated effects of hypothetical excise tax increases in Senegal and Nigeria for married smokers.

Increase of Prices (Increase of Taxes)	Relative Decline of the Demand		Relative Change of Tax Revenues	
	Senegal	Nigeria	Senegal	Nigeria
5 (6.66)	-7.45	-0.83	-2.82	4.12
10 (13.33)	-14.90	-1.66	-6.39	8.17
15 (20)	-22.35	-2.49	-10.70	12.13
20 (26.66)	-29.80	-3.32	-15.76	16.01
25 (33.33)	-37.25	-4.15	-21.56	19.81
30 (40)	-44.70	-4.98	-28.11	23.52
35 (46.66)	-52.15	-5.81	-35.40	27.15
40 (53.33)	-59.60	-6.64	-43.44	30.70
45 (60)	-67.05	-7.47	-52.22	34.16
50 (66.66)	-74.50	-8.3	-61.75	37.55
60 (80)	-89.40	-9.96	-83.04	44.06
67.11 (89.48)	-100	-11.14	-99.98	48.49
70 (93.33)	…	-11.62	…	50.24
80 (106.66)	…	-13.28	-83.52	56.09
90 (120)	…	-14.94	…	61.61
100 (133.33)	…	-16.60	…	66.80
…	…	…	…	…
251.20 (334.93)	…	-41.69	…	104.75
…	…	…	…	…
502.40 (669.86)	…	-83.39	…	0.00

All the figures in the table are expressed in percentage terms. Price-elasticities of demand: -1.49 for Senegal and -0.166 for Nigeria.

**Table 3:** Simulated effects of hypothetical excise tax increases in Senegal and Nigeria for male smokers.

Increase of Prices (Increase of Taxes)	Relative Decline of the Demand		Relative Change of Tax Revenues	
	Senegal	Nigeria	Senegal	Nigeria
5 (6.66)	-6.65	-1.01	-1.98	3.94
10 (13.33)	-13.30	-2.01	-4.63	7.78
15 (20)	-19.95	-3.01	-7.94	11.53
20 (26.66)	-26.60	-4.02	-11.92	15.17
25 (33.33)	-33.25	-5.02	-16.56	18.71
30 (40)	-39.90	-6.03	-21.87	22.16
35 (46.66)	-46.55	-7.03	-27.84	25.50
40 (53.33)	-53.20	-8.04	-34.48	28.74
45 (60)	-59.85	-9.04	-41.78	31.88
50 (66.66)	-66.50	-10.05	-49.75	34.92
60 (80)	-79.80	-12.06	-67.68	40.70
70 (93.33)	-93.10	-14.07	-88.27	46.08
75.18 (100.24)	-100	15.11	-99.98	48.70
80 (106.66)	…	-16.08	…	51.05
90 (120)	…	-18.09	…	55.62
100 (133.33)	…	-20.10	…	59.80
…	…	…	…	…
198.75 (265)	…	-39.94	…	79.40
…	…	…	…	…
397.51 (530)	…	-79.89	…	0.00

All the figures in the table are expressed in percentage terms. Price-elasticities of demand: -1.33 for Senegal and -0.201 for Nigeria.

**Table 4:** Simulated effects of hypothetical excise tax increases in Senegal and Nigeria for rural smokers.

Increase of Prices (Increase of Taxes)	Relative Decline of the Demand		Relative Change of Tax Revenues	
	Senegal	Nigeria	Senegal	Nigeria
5 (6.66)	-8.35	-1.13	-3.76	3.81
10 (13.33)	-16.70	-2.26	-8.37	7.51
15 (20)	-25.05	-3.39	-13.80	11.10
20 (26.66)	-33.40	-4.52	-20.08	14.57
25 (33.33)	-41.75	-5.65	-27.18	17.93
30 (40)	-50.10	-6.78	-35.13	21.18
35 (46.66)	-58.45	-7.91	-43.90	24.32
40 (53.33)	-66.80	-9.04	-53.52	27.34
45 (60)	-75.15	-10.17	-63.96	30.25
50 (66.66)	-83.50	-11.30	-75.25	33.05
59.88 (79.84)	-100	-13.53	-99.99	38.34
60 (80)	…	-13.56	…	38.30
70 (93.33)	…	-15.82	…	43.10
80 (106.66)	…	-18.08	…	47.45
90 (120)	…	-20.34	…	51.35
100 (133.33)	…	-22.60	…	54.80
…	…	…	…	…
171.233 (228.30)	…	-38.69	…	66.26
…	…	…	…	…
342.47 (456.61)	…	-77.39	…	0.00

All the figures in the table are expressed in percentage terms. Price-elasticities of demand: -1.67 for Senegal and -0.226 for Nigeria.

## Results

### Estimation of the price-elasticities

Before commenting on the results of the simulations, it is first important to shed some light on the results of the estimation of the price-elasticities of tobacco demand *ϵ*_*p*_. These estimations stem from the GATS datasets, provided by S1 and S2 Datatsets. The price-elasticities of demand are calculated for the entire sample first and then for each sub-group of cigarettes users: men, women, different age groups, rural and urban populations. Some estimated elasticities are not really significant and therefore would not be included in our analysis. In general, two criteria have been retained in the selection process of the price-elasticities to be used in the analysis: (a) only the highly significant estimates of the price-elasticities (between 1 and 5% degree of significance) would be used in the simulation; (b) among those estimates, we will only focus on the sub-groups for which the price-elasticities of tobacco demand are available for both Senegal and Nigeria. These criteria are without a general loss of generality and imply that we will have to simulate the effects of tax increases on the prices, the demand and the tax revenues for four categories of tobacco users in each of the two countries considered: men, tobacco users living in rural areas, married smokers as well as the entire population of tobacco users.

The GATS data are cross-sectional; thus, the price of cigarettes is derived from the prices that current smokers reported for their last purchase. We construct a weighted average price from self-reported prices in the survey. The level of aggregation is either regional or at the smallest unit of sampling. For Senegal, the price variable is at the regional level, while for Nigeria the level of aggregation is the primary level of sampling defined during their last census in 2006. In order to account for the effect of wealth on cigarettes consumption, we construct a wealth index of the individuals’ household. Although, the GATS does not report the income or the households’ consumption expenditures, different items such as television, bicycle, car and other assets owned by the households are reported. We follow the methodology of the World Health Organization and adopt a principal component analysis. This method generates a continuous scale of wealth based on the items owned by the households. All variables reporting the different items are recorded in binary variables. The value 1 is assigned when the household owns one particular item and zero otherwise. The list of the variables and their definition are given in S1 Appendix, while S2 Appendix provides the descriptive statistics for Senegal and Nigeria.

The Heckman selection model used to estimate the price-elasticities provides the possibility to correct the sample selection bias due to the fact that the current smokers are a sub-sample of the collected sample. In a situation whereby, the variable of response is not observed for the whole population, it is necessary to correct for the resulting bias. In the GATS data, the quantity of smoked cigarettes per day is not reported for non smokers. These missing observations correspond to a potential variable of response and therefore the model of selection of Heckman is more appropriate. We thus adopt that model for the estimation of the price-elasticity of the demand. Generally, the Heckman selection model (or Tobit model of type 2) is as follows.

1. A selection equation (Latent Probit model):
  $$\begin{array}{c} T_{i}=1_{\left[T_{i}^{*}>0\right]} \end{array}$$ (16)
  With
  $$\begin{array}{c} T_{i}^{*}=\gamma^{\prime }w_{i}+\mu_{i} \end{array}$$ (17)
  Where $w_{i}^{*}$ is the row vector of the control variables; **1**_[.]_ the indicator function taking the value 1 in case of the realization of the event [.]; *γ* the vector of the parameters to estimate and *μ*_*i*_ the error term with a normal distribution $N(0,\delta_{\mu }^{2})$. We suppose that **w**_*i*_ ⊥ *μ*_*i*_.
2. An equation of intensity of the consumption of cigarettes (linear regression):
  $$\begin{array}{c} y_{i}=\beta^{\prime }x_{i}+\delta T_{i}+\varepsilon_{i} \end{array}$$ (18)
  Where **x**_*i*_ is the row vector of the control variables, ***β*** and *δ* are the vectors of parameters to estimate, and *ε*_*i*_ the error term. Since *T*_*i*_ is endogeneous, **E**(*ε*_*i*_|*T*_*i*_) ≠ 0.

The control variables in the two equations are the price, the gender, the living area (urban or rural), the age groups, the level of education, the socio-economic status—measured by the household’s wealth –, the employment status. For a better identification of the selection model, it is necessary to have at least one variable that affects the selection and not the response variable in the equation of intensity. Such variables are called exclusion restriction variables. Accordingly, in the selection equation we add another variable that only affect the choice to smoke or not. The main requirement for a restriction variable is that it indirectly affects the main response variable, in this case the quantity of cigarettes consumed. The restriction variable that we use to estimate the Heckman selection model is the proscription of smoking by religion. This variable fulfills the necessary criteria since it is reported for all individuals in the survey and is strongly related to the probability of smoking. Regarding the variable prohibition of smoking by the religion of the individual, it certainly affects the decision of individuals to smoke or not. This variable is most likely to affect the decision to participate than the quantity consumed. The quantity of cigarettes smoked per day and the price variable are transformed in logarithm. Two methods are generally adopted in the literature for the estimation of models (17) and (18): the Full Information Maximum Likelihood (FIML) and the two-steps method of Heckman. We chose to apply both. The estimation results of the price-elasticities of cigarettes’ demand for Senegal and Nigeria are all consigned in S3 Appendix.

The estimations, available in S3 Appendix, give -1.29 and -0.217 as price-elasticities of tobacco demand for all tobacco users in Senegal and Nigeria respectively. Equally, -1.49 and -0.166 are the price-elasticities of tobacco demand for married smokers in Senegal and Nigeria respectively. In addition, -1.33 and -0.201 are the price-elasticities of demand for male smokers in Senegal and Nigeria respectively. Finally, the price-elasticities of tobacco demand for tobacco users living in rural areas are -1.67 and -0.226 for Senegal and Nigeria respectively. The negative signs attached to these elasticities show that despite its addictive nature, cigarette remains a normal good, given that its price increases reduce its short-term demand. More specifically, these numbers suggest for example that a 10% increase of the prices reduces the consumption of cigarettes for all smokers by 12.9% in Senegal and by 2.17% in Nigeria. The interpretation is the same for the remaining elasticities.

The first takeaway from these estimates is the very low level of the price-elasticity of tobacco demand in Nigeria and the high level of price-elasticity of tobacco demand in Senegal, no matter which sub-group of tobacco users considered. This strong inelasticity of demand in the former country justifies otherwise the advocacy by some specialists of a dramatic increase of the price of tobacco, through an ambitious taxation policy ([11, 15]). The second lesson is that, among all the sub-groups of tobacco users considered, the ones living in rural areas in Senegal are those with the highest sensitivity to price changes in the country, while the smokers with the marital status ‘married’ in Nigeria are the least sensitive to price variations, compared to the rest of the population.

### Results of the tax simulation

#### Entire population

Table 1 presents the results of the tax simulation for the entire population of tobacco users in both Senegal and Nigeria. The first column shows the hypothetical successive price increases—all things being equal—ranging from 5,10,15 to 100% and beyond. The initial tax increases that triggered the changes in prices are in parentheses. Every one unit increase of the excise taxes leads to a 0.75 unit increase of the prices. All the numbers present in the table are expressed in percentage terms. Columns 2 and 3 indicate the relative decline in the demand following the tax increase for Senegal and Nigeria respectively. Columns 4 and 5 report the relative variations of the tax revenues for Senegal and Nigeria respectively, as a result of successive tax increases (and therefore price increases). It mainly appears from the simulation exercise that tax development does not have the same implication for Senegal and Nigeria, especially when the tax revenues are concerned. This discrepancy stems from the differences between the estimated price-elasticities of demand.

Without a major surprise—and as somehow initially expected—increasing taxes (hence prices) implies a reduction of the cigarettes demand in the two countries. However, the decrease in demand is faster in Senegal than in Nigeria. This comes from the fact that the estimates of the price-elasticities of cigarettes demand are quite high in the former country compared to the latter. For example, a 20% price increase on tobacco, as the result of a 26.66% of tax increase, implies a 25.8% decline of the demand in Senegal and of only 4.34% in Nigeria. To reduce by half the demand of cigarettes, it “suffices” to increase the taxes in the vicinity of 60% in Senegal, therefore the prices by nearly 45%. To reach the same result in Nigeria, prices need to be increased by more than 200%.

If the relative variations of cigarettes demand in Senegal and Nigeria go along in the same direction (i.e., a reduction) following an increase in prices, such is not exactly the case when the tax revenues are concerned. While the successive tax increases (hence price increases) in Nigeria successively increase the tax revenues, they however reduce those tax revenues in the case of Senegal. This partly comes from very high value of the price-elasticity of tobacco demand in Senegal. For example, while a 10% increase of prices, resulting from a 13.33% tax increase, induces an 12.9% decline of the cigarettes demand in Senegal. This same move could however imply a 2.17% contraction of the tax revenues in the country. The argument is quite different for Nigeria. The increase of taxes (hence of prices) in the country induces an increase of the tax revenues. For example, increasing the prices by 10%, as a result of a 13.33% tax increase, leads to a 7.61% hike in the tax revenues in Nigeria.

However, the increase in tax revenues is not limitless when Nigeria is concerned. From a certain threshold value of the excise tax, the revenues entirely cease to increase before starting to steadily decline. The threshold beyond which the tax increases do not positively affect the tax revenues in Nigeria anymore is 240.54%, which corresponds to 180.41% of prices. An increase of taxes (hence of prices) corresponding to this value induces a 70.63% increase of the tax revenues—the maximum possible—while the demand would at most decline by 39.14%. Equally, an aggressive tax policy of 481.09% would lead to a 360.82% price hike and to a 78.29% decline in the demand of cigarettes, while keeping the tax revenues unchanged. This situation corresponds to the moment when the new tax revenues generated from the price increase exactly compensate the losses in tax revenues thanks in part to the decline in the number of tobacco users. The numbers 240.54% and 481.09% represent the critical values of the tobacco tax policies in Nigeria.

#### Results for sub-groups of smokers

Tables 2–4 replicate the same simulations, but this time for tobacco users who happen to be married, for the male smokers and for those living in rural areas, both in Senegal and Nigeria respectively. By briefly comparing the price-elasticities among the different sub-groups, we observe more or less a certain degree of homogeneity. However, the tobacco users living in rural Senegal are more sensitive than the other categories of smokers. This difference could stem from the revenue discrepancy between rural and urban populations. This implies for instance that for an equal price increase, the demand of tobacco users in Senegal living in rural areas decreases way faster than those who live in non-rural areas. In Nigeria, the only noticeable observation is the lower value of the price-elasticity of tobacco demand for the married smokers. Notwithstanding these differences, the interpretations of the various outcomes of the simulations as well as the evolution pattern of the cigarettes demand and that of the tax revenues generally remain the same.

## Conclusion

Cigarette consumption has long been acknowledged as a major public health issue, especially when the users are teenagers and pregnant women. Cigarette has caused a heavy death toll, since it became a public consumption good in the middle of the 19^*th*^ century. According to the World Health Organization (WHO), the tobacco epidemic alone was responsible of the death of 100 million people throughout the 20^*th*^ century. And if things keep following a business as usual course, that figure could eventually reach the billion by the end of the ongoing century, while particularly affecting developing countries such as Senegal and Nigeria. In that regard, the WHO since many years now already collaborates with all its member states for the implementation of the Framework Convention on Tobacco Control (FCTC), especially by working with the ministers of finance of these countries to help them design and implement comprehensive tobacco taxation policies. For all countries concerned, a fundamental consideration is the capacity to generate enough fiscal revenues on a relatively long period of time. This paper uses a double approach to infer on the evolution of prices first and that of the demand as well as that of the tax revenues both in Senegal and Nigeria. The theoretical model predicts the prevalence of the specific excise taxes on the ad valorem ones for Senegal and the prevalence of the latter on the first in Nigeria. Furthermore, in a market with different varieties of a good, the degree of market concentration is directly a function of the price-elasticity of demand of that good. The results of the theoretical model also show that in the context of excise taxation, the number of varieties—or of brands—directly affects both the degree of market concentration and the marginal effect of specific and ad valorem excise taxes on the price of tobacco. In addition, the comparison of the marginal effects of ad valorem and specific excise taxes depends on the marginal costs of production of different varieties weighted by the tax rates and the number of varieties.

Regarding the simulation model, and relying mainly on data from the Global Adult Tobacco Survey (GATS) for Senegal and Nigeria, this research intended to show that tobacco excise taxes increases reduce substantially more the demand of cigarettes in Senegal than in Nigeria. However, these tax increases only positively affect the government revenues in Nigeria. This difference stems from the fact that the price-elasticity of demand is very high in Senegal, contrary to Nigeria. Equally, among all the categories considered, the smokers living in rural areas in Senegal are more sensitive to price changes, while in Nigeria the married smokers are less sensitive than the rest of the population to changes in prices. Finally, even in Nigeria where the tax revenues follow the expected trend, these successive increases are not unlimited. There is a certain specific threshold beyond which the tax revenues cease to increase.

More specifically, the specific excise taxes seem more appropriate for Senegal while ad valorem excise taxes seem best for Nigeria. These results stem from the predictions of the product-variety model, which extends [11] and [12]. Given that price represents the major determinant of demand, it is important for Senegal and Nigeria to give more importance to specific and ad valorem excise taxes respectively. The idea is not to entirely substitute the ad valorem excise taxes by the specific ones or vice versa. It is entirely possible to build a situation where both forms of excise taxes coexist, which is not entirely the case at the moment. In 2016 for example, the rates of ad valorem excise taxes in Senegal and Nigeria were respectively 22.5 and 15.87%, while there was nearly no specific excise tax on retailed cigarettes in both of these countries [14].

While the double objective of simultaneously developing public tax revenues and reducing tobacco demand is perfectly doable in Nigeria, such policy is not possible in Senegal given all the current circumstances. This mostly stems from the fact that the price-elasticity of tobacco demand is quite high in the latter country (-1.29). Therefore, the Senegalese government should consent to give up on a certain fraction of its current excise tax revenues if it intends to further lower the consumption of cigarettes. A tax increase for all the tobacco users of 53% in Senegal would lead to a 40% price hike and to a decline by nearly half of the tobacco demand in the country. This move however could imply a 32% decline of the tax revenues.

Given the very low value of the price-elasticity of cigarettes demand in Nigeria (-0.217), the authorities in this country seem to have a higher flexibility, contrary to their counterparts in Senegal. In Nigeria, the objective of the tobacco demand reduction is not compatible with that of further mobilization of additional tax revenues. The country could therefore pursue an aggressive taxation policy in order to control the demand of cigarettes. More specifically, the Nigerian authorities could increase the current tobacco taxes by up to 240% before reaching a threshold beyond which tax revenues cease to increase with respect to their previous values. Such development could lead to a 180% increase of the price and to a 70% of the tax revenues. Beyond that threshold however, the tax revenues begin to decline steadily. An aggressive taxation policy consisting of increasing the taxes by 481% in Nigeria could help reduce the cigarettes demand by 78%, while keeping unchanged the current level of tax revenues.

Finally, it is possible for each one of these two countries to put in place a differentiated taxation scheme, mainly based on the geographical location. A 60% tax increase in the rural areas in Senegal could reduce the demand by up to 75%, while a similar increase “only” reduces the demand for all smokers by 58%. It could therefore be possible to tax the consumers living in rural areas differently from the rest of the population and reaching the same outcomes. Equally, increasing taxes by 60% on rural smokers in Nigeria would lower the demand by 10%, while increasing the overall tax revenues by 30%. However, setting up a differentiated taxation scheme could face two major stumbling blocks: the incompatibility with the legislative frameworks in place and the problem of smuggling. Taxing differently many groups of tobacco users could be at variance with Senegalese and Nigerian legislation. In addition, differentiated prices by geographical regions could exacerbate the problem of cigarettes smuggling.

All these recommendations stem from our early simulations, of which the key ingredient is the price-elasticity of cigarettes demand in Senegal and in Nigeria. The very low value of the price-elasticity in Nigeria, no matter which category of consumers is considered, testifies for a strong inelasticity of tobacco demand as a result of changes in price in this country, as already observed by [40]. On the contrary, the very high price-elasticity of demand in Senegal could suggest that taxation might have reached its critical threshold, given the local circumstances, or simply that consumers in Senegal are more exposed to various substitutes to tobacco. It is also important to remember that all simulations derive from the hypothesis that a one unit tax increase induces a 0.75 increase of the tobacco price, a departure from the one-to-one relationship between taxes and prices, an assumption used for example by [4] and [2] among others.

## Supporting information

S1 Dataset Global Adult Tobacco Survey (GATS) data for Senegal. (DTA)

S2 Dataset Global Adult Tobacco Survey (GATS) data for Nigeria. (DTA)

S1 File Stata estimation codes for the price-elasticities. (DO)

S1 Appendix List and definition of variables. (PDF)

S2 Appendix Descriptive statistics for Senegal and Nigeria. (PDF)

S3 Appendix Estimation results for Senegal and Nigeria. (PDF)
