# Supplementary material for: Public finances and tobacco taxation with product variety: Theory and application to Senegal and Nigeria
Source: PLoS One. 2019 Feb 14;14(2):e0212015. doi: 10.1371/journal.pone.0212015 (PMC6375595; doi:10.1371/journal.pone.0212015)
Supplement: S1 Appendix — (PDF) [file pone.0212015.s004.pdf]

Table 1. List and definition of variables

**Variables**	**Definition**	**Nature**
Consumption per day	Number of smoked cigarettes per day	Continuous
Smoking prevalence	Whether the individual smokes	Binary
Price	Weighted average price of cigarettes	Continuous
Male	Gender of the individual	Binary (Male=1)
Age 15-24	Individual is aged between 15 and 24	Binary
Age 25-44	Individual is aged between 25 and 44	Binary (reference)
Age 45-64	Individual is aged between 45 and 64	Binary
Age 65+	Individual is aged 64 or more	Binary
Urban area	Living in an urban area	Binary
Without education	Individual with no level of education	Binary
Elementary education	Elementary level of education reached	Binary
Secondary education	Secondary level of education reached	Binary (reference)
University education	University level of education reached	Binary
Wealth	Wealth index of the individual' s household	Continuous
Employee	Individual working for others	Binary
Independent	Self-employed individual	Binary (reference)
Inactive	Individual who is not working neither looking for job	Binary
Unemployed	Individual who is not working but looking for job	Binary
Single	Individual who is not engaged	Binary
Married	Individual who is married	Binary (reference)
Divorced/separated	Individual who no longer have a partner	Binary
Widow	Individual whose partner is deceased	Binary
Prohibited by the religion	Prohibition of smoking by the individual's religion	Binary
