# Supplementary material for: Public finances and tobacco taxation with product variety: Theory and application to Senegal and Nigeria
Source: PLoS One. 2019 Feb 14;14(2):e0212015. doi: 10.1371/journal.pone.0212015 (PMC6375595; doi:10.1371/journal.pone.0212015)
Supplement: S2 Appendix — (PDF) [file pone.0212015.s005.pdf]

Table 1. Descriptive statistics for Senegal

Variables	#Obs	Average	Standard deviation [a]	Min[b]	Max[c]
**Dependent Variables**					
Cigarettes prevalence	4343	0.061			
Daily consumption of sticks	265	5.942	7.193	0	40
**Control variables**					
Price in FCFA	4343	29.466	5.293	19.443	41.049
Male	4343	0.451			
Age 15-24	4343	0.274			
Age 25-44	4343	0.447			
Age 45-64	4343	0.216			
Age 65+	4343	0.063			
Urban area	4343	0.506			
Without education	4336	0.571			
Elementary education	4336	0.193			
Secondary education	4336	0.193			
University education	4336	0.042			
Wealth index	4326	-0.151	1.910	-2.583	8.436
Employee	4334	0.107			
Independent	4334	0.409			
Inactive	4334	0.418			
Unemployed	4334	0.066			
Single	4343	0.282			
Married	4343	0.643			
Divorced/separated	4343	0.022			
Widow	4343	0.053			
Prohibited by the religion	4239	0.959			

[a,b,c] Standard deviations, Min. and Max. are not reported for the binary variables.

Table 2. Descriptive statistics for Nigeria

Variables	#Obs	Average	Standard deviation [a]	Min[b]	Max[c]
**Dependent variables**					
Cigarettes prevalence	9705	0.036			
Daily consumption of sticks	350	5.684	5.545	0.143	40
**Control variables**					
Price in Naira	9705	12.455	10.065	2	48.687
Male	9705	0.516			
Age 15-24	9704	0.221			
Age 25-44	9704	0.506			
Age 45-64	9704	0.193			
Age 65+	9704	0.079			
Urban area	9705	0.492			
Without education	9693	0.319			
Elementary education	9693	0.196			
Secondary education	9693	0.348			
University education	9693	0.136			
Wealth index	9649	-0.021	0.909	-0.119	8.369
Employee	9696	0.132			
Independent	9696	0.542			
Inactive	9696	0.283			
Unemployed	9696	0.043			
Single	9696	0.261			
Married	9696	0.638			
Divorced/separated	9696	0.029			
Widow	9696	0.072			
Prohibited by religion	9526	0.968			

[a,b,c] Standard deviations, Min. and Max. are not reported for the binary variables.
