# Supplementary material for: Public finances and tobacco taxation with product variety: Theory and application to Senegal and Nigeria
Source: PLoS One. 2019 Feb 14;14(2):e0212015. doi: 10.1371/journal.pone.0212015 (PMC6375595; doi:10.1371/journal.pone.0212015)
Supplement: S3 Appendix — (PDF) [file pone.0212015.s006.pdf]

Table 1. Estimation of cigarettes' demand for Senegal

Variables	FIML		Two-step	
	Coef.	Err.	Coef.	Err.
**Consumption**				
Price	-1.294***	0.499	-1.263**	0.497
Male	-0.268	0.437	-0.371	0.722
Age group(ref. Age 25-44)				
Age 15-24	0.163	0.225	0.187	0.261
Age 45-64	-0.350**	0.156	-0.358**	0.160
Age 65+	-0.077	0.476	-0.062	0.386
Urban area	0.368**	0.172	0.356**	0.172
Education (ref. secondary)				
No education	-0.223	0.231	-0.218	0.218
Primary	-0.305	0.235	-0.317	0.225
University	0.382	0.313	0.372	0.348
Wealth Index	0.123**	0.056	0.129**	0.063
Employment(ref. self-employed)				
Employee	0.161	0.206	0.170	0.202
Inactive	-0.271	0.264	-0.255	0.254
Unemployed	-0.078	0.297	-0.078	0.265
Marital status (married)				
Single	-0.214	0.156	-0.213	0.166
Divorced	0.061	0.341	0.021	0.389
Widowed	-0.473	0.357	-0.467	0.819
Intercept	6.804***	1.578341	6.943***	1.753422

Continued on next page...

Table 2. Estimation of cigarettes' demand for Senegal: continued

Variables	FIML		Two-step	
	Coef.	Err.	Coef.	Err.
**Smoking participation**$^{(a)}$				
Price	-0.023**	0.01	-0.023**	0.011
Male	0.068***	0.007	0.068***	0.007
Age group(ref. Age 25-44)				
Age 15-24	-0.019***	0.006	-0.019***	0.006
Age 45-64	0.004	0.004	0.004	0.004
Age 65+	-0.017*	0.008	-0.017*	0.009
Urban area	0.008**	0.004	0.008*	0.004
Education (ref. secondary)				
No education	-0.004***	0.001	-0.004***	0.001
Primary	-0.001	0.006	-0.001	0.006
University	0.010*	0.006	0.010*	0.006
Wealth Index	0.011	0.009	0.011	0.009
Employment(ref. self-employed)				
Employee	-0.007	0.005	-0.007	0.005
Inactive	-0.010*	0.006	-0.010*	0.005
Unemployed	0.000	0.007	0.000	0.007
Marital status (married)				
Single	0.000	0.005	0.000	0.005
Divorced	0.027***	0.01	0.027***	0.009
Widowed	0.004	0.015	0.004	0.014
Prohibited by religion	-0.026***	0.007	-0.026***	0.007
# Observations	4208			
# Censured observations	3956			
# Non censured observations	252			
$\chi_2(16)$	102.05		61.24	
p-value	0.000		0.000	
Log Likelihhod	-1095.869			
Mills' ratio			-0.4967979	0.5131982
$\rho = cov(\mu_i, \varepsilon_i)$	-0.4093362	0.3078213		
Wald test H_0: ρ=0				
Wald $\chi_2(1)$	0.29			
Prob > χ_2	0.1836			
σ_μ	1.061725	0.1112047		
$\lambda = \sigma_\mu \rho$	-0.4118527	0.3184603		

$^{(a)}$ Marginal effects.

Significance level: $*$: 10% $**$: 5% $***$: 1%

Table 3. Estimation of cigarettes' demand for Nigeria

Variables	FIML		Two-step	
	Coef.	Err.	Coef.	Err.
**Consumption**				
Price	-0.217***	0.065	-0.211***	0.070
Male	-0.298	0.373	-0.207	0.499
Age group(ref. Age 25-44)				
Age 15-24	-0.252	0.170	-0.275	0.212
Age 45-64	-0.022	0.107	-0.028	0.107
Age 65+	-0.103	0.164	-0.121	0.235
Urban area	-0.205*	0.120	-0.235	0.165
Education (ref. secondary)				
No education	0.066	0.130	0.076	0.134
Primary	-0.019	0.120	-0.003	0.135
University	-0.005	0.150	-0.011	0.148
Wealth index	0.052*	0.029	0.054*	0.032
Employment(ref. self-employed)				
Employee	0.229*	0.123	0.237*	0.126
Inactive	-0.130	0.198	-0.160	0.252
Unemployed	0.073	0.222	0.073	0.258
Marital status (married)				
Single	0.129	0.110	0.128	0.115
Divorced	-0.009	0.147	0.013	0.199
Widowed	0.086	0.379	0.067	0.447
Intercept	2.286***	0.608	2.101**	0.914

Continued on next page...

Table 4. Estimation of cigarettes' demand for Nigeria: continued

Variables	FIML		Two-step	
	Coef.	Err.	Coef.	Err.
**Smoking participation**[a]				
Price	0.008*	0.005	0.008	0.005
Male	0.153***	0.012	0.153***	0.012
Age group(ref. Age 25-44)				
Age 15-24	-0.043***	0.014	-0.043***	0.014
Age 45-64	-0.008	0.009	-0.008	0.009
Age 65+	-0.037**	0.016	-0.037**	0.017
Urban area	-0.054***	0.009	-0.054***	0.008
Education (ref. secondary)				
No education	0.003	0.003	0.003	0.003
Primary	0.015	0.01	0.015	0.01
University	0.026***	0.01	0.026***	0.01
Wealth Index	-0.012	0.011	-0.012	0.011
Employment(ref. self-employed)				
Employee	0.01	0.01	0.01	0.01
Inactive	-0.037***	0.014	-0.037***	0.014
Unemployed	0.004	0.019	0.004	0.019
Marital status (married)				
Single	-0.007	0.01	-0.007	0.01
Divorced	0.032**	0.015	0.032**	0.016
Widowed	-0.029	0.026	-0.029	0.029
Prohibited by religion	-0.055***	0.015	-0.055***	0.015
#Observations	2772			
# Censured observations	2450			
# Non censured observations	322			
$\chi_2(16)$	38.09		30.34	
p-value	0.0015		0.0163	
Log Likelihod	-1104.095			
Mills ratio			0.1734887	0.3289036
$\rho = cov(\mu_i, \varepsilon_i)$	0.1417713	0.261912		
Wald test H_0: ρ=0				
$\chi_2(1)$	0.5883			
Prob > χ_2	0.1836			
σ_μ	0.7284151	0.0316926		
$\lambda = \sigma_\mu \rho$	0.102582	0.1896428		

[a] Marginal effects.

Significance level: $*$: 10% $**$: 5% $***$: 1%

Table 5. Price-elasticity of cigarettes' demand in Senegal

Group	FIML		Two-step	
	Elasticity	Err.	Elasticity	Err.
Average	-1.29***	0.50	-1.26 **	0.50
Age 15-24	...	...	2.67	4.52
Age 25-44	-0.91	0.56	-1.01	0.73
Urban	-0.58*	0.80	-0.58	0.70
Rural	-1.67 ***	0.57	-1.26	0.86
Male	-1.33***	0.50	-1.27**	0.51
Without education	-1.89**	0.78***	-1.90**	0.68
Single	-0.52	1.03	-0.52	1.02
Married	-1.49**	0.62	-1.49**	0.61
Lowest level of wealth	...	...	-2.10*	0.62

Table 6. Price-elasticity of cigarettes' demand in Nigeria

Group	FIML		Two-step	
	Elasticity	Err.	Elasticity	Err.
Average	-0.217***	0.065	-0.211***	0.070
Age 15-24	...	...	-0.626***	0.201
Age 25-44	-0.157 *	0.081	-0.150*	0.084
Urban	-0.212*	0.123	-0.212*	0.123
Rural	-0.226***	0.072	-0.217 ***	0.088
Male	-0.201***	0.070	-0.190***	0.077
No education	-0.084	0.141	-0.197	0.313
Single	-0.232	0.151	-0.194	0.195
Married	-0.166**	0.080	-0.167**	0.083
Lowest level of wealth	-0.279**	0.135	-0.211***	0.070
